# Advancing cancer theranostics through biomimetics: A comprehensive review

**DOI:** 10.1016/j.heliyon.2024.e27692

**Published:** 2024-03-11

**Authors:** Kuttiappan Anitha, Santenna Chenchula, Vijayaraj Surendran, Bhatt Shvetank, Parameswar Ravula, Rhythm Milan, Radhika Chikatipalli, Padmavathi R

**Affiliations:** a Department of Pharmacology, School of Pharmacy and Technology Management (SPTM), SVKM's Narsee Monjee Institute of Management Studies (NMIMS) Deemed-to-University, Shirpur, 425405, India; b Department of Clinical Pharmacology, All India Institute of Medical Sciences (AIIMS), Bhopal, 462020, Madhya Pradesh, India; c Dr Kalam College of Pharmacy, Thanjavur District, Tamil Nadu, 614 623, India; d School of Health Sciences and Technology, Dr Vishwanath Karad MIT World Peace University, Pune, 411038, Maharashtra, India; e Amity Institute of Pharmacy, Amity University Madhya Pradesh (AUMP), Gwalior, 474005, Madhya Pradesh, India; f Sri Venkateshwara College of Pharmacy, Chittoor District, Andhra Pradesh, 517520, India; g SVS Medical College, Mahbubnagar, Telangana, India

**Keywords:** Theranostics, Biomimetics, Lung cancer, Breast cancer, Skin cancer, Gastric cancer

## Abstract

Nanotheranostics, especially those employing biomimetic approaches, are of substantial interest for molecular imaging and cancer therapy. The incorporation of diagnostics and therapeutics, known as cancer theranostics, represents a promising strategy in modern oncology. Biomimetics, inspired by nature, offers a multidisciplinary avenue with potential in advancing cancer theranostics. This review comprehensively analyses recent progress in biomimetics-based cancer theranostics, emphasizing its role in overcoming current treatment challenges, with a focus on breast, prostate, and skin cancers. Biomimetic approaches have been explored to address multidrug resistance (MDR), emphasizing their role in immunotherapy and photothermal therapy. The specific areas covered include biomimetic drug delivery systems bypassing MDR mechanisms, biomimetic platforms for immune checkpoint blockade, immune cell modulation, and photothermal tumor ablation. Pretargeting techniques enhancing radiotherapeutic agent uptake are discussed, along with a comprehensive review of clinical trials of global nanotheranostics. This review delves into biomimetic materials, nanotechnology, and bioinspired strategies for cancer imaging, diagnosis, and targeted drug delivery. These include imaging probes, contrast agents, and biosensors for enhanced specificity and sensitivity. Biomimetic strategies for targeted drug delivery involve the design of nanoparticles, liposomes, and hydrogels for site-specific delivery and improved therapeutic efficacy. Overall, this current review provides valuable information for investigators, clinicians, and biomedical engineers, offering insights into the latest biomimetics applications in cancer theranostics. Leveraging biomimetics aims to revolutionize cancer diagnosis, treatment, and patient outcomes.

## Highlights

• Nanotheranostics are of substantial interest for molecular imaging and cancer therapy.
• Recent developments in cancer nanotranostics have been reviewed.
• Biomimetic drug delivery systems for addressing multidrug resistance (MDR) and enhancing drug penetration into tumor tissues have been explored.
• Biomimetic platforms for immune checkpoint blockade, immune cell modulation, and photothermal tumor ablation are discussed.
• We reviewed the investigations on how pretargeting techniques enhance the uptake and retention of radiotherapeutic agents at the target site.

## 1 Introduction

Tumor treatment remains one of most formidable encounters in modern medicine, necessitating innovative approaches that transcend traditional therapeutic paradigms. The emergence of theranostics, a field that combines diagnostics and therapeutics, has ushered in a new era of personalized, targeted cancer management [1]. This paradigm shift has paved the way for advancements that go beyond conventional methodologies, aiming to optimize treatment outcomes while minimizing adverse effects. In this comprehensive review, we delve into the intersection of cancer theranostics and biomimetics, exploring how nature-inspired design principles hold the key to overcoming current challenges in cancer diagnosis and treatment [2].

### 1.1 Advancements in medicine: basics of cancer theranostics

The word "theranostic" was coined by John Funkhouser and embodies a revolutionary concept in medical sciences that aligns diagnostics and therapeutics into a singular, integrated entity. This integration facilitates a targeted and personalized approach to treatment, emphasizing the critical interplay between diagnosis, drug delivery, and treatment response monitoring [[3], [4], [5]]. Traditional medicine, rooted in historical justifications, has evolved into a diverse spectrum of medical modalities that now incorporate chemical compounds from various sources. The transition from reactive practices, focusing on clinical signs and symptoms, to proactive strategies involving genetic profiling and advanced imaging techniques marks a significant stride in medical progress [6].

The evolution of medicine, particularly in the field of oncology, has witnessed a shift from trial-and-error medication to personalized approaches. Theranostics, as a close collaboration between diagnostics and therapeutics, play a pivotal role in tailoring medication treatments based on individual genetic profiles [7,8]. This approach holds promise for reducing time and cost, increasing success rates, and guiding preclinical medicine development or clinical trial eligibility.

### 1.2 Theranostics: revolutionizing personalized medicine

Since its inception, theranostics have revolutionized the landscape of targeted and individualized therapies. The ability to combine treatment and diagnostics offers a unique avenue for delivering personalized medicine that considers complex relationships, genetic variations, and environmental influences on treatment responses. The financial burden of ineffective treatments, which accounts for a significant portion of the global pharmaceutical industry, underscores the urgency of developing specific and targeted therapies [9]. The disruptive growth of cancer cells, especially in diseases such as breast cancer, prostate cancer, and skin cancer, demands innovative strategies beyond traditional treatment modalities [10].

The amalgamation of theranostics with nanotechnology has introduced new directions for cancer treatment. Nanomaterials, including liposomes, dendrimers, metallic nanoparticles, and quantum dots, present multifunctional platforms for cancer theranostics [[11], [12], [13]]. These materials, with their unique properties, enable simultaneous diagnosis and therapy, offering opportunities for enhanced therapeutic efficacy and reduced adverse effects. This comprehensive review explores these advancements, shedding light on the potential of nanotheranostics to address challenges associated with conventional cancer treatments.

### 1.3 Nano-theranostic advancements

Nanorevolution in medicine has propelled the development of nanotheranostics, presenting a range of advantages from bypassing first-pass metabolism to achieving systemic circulation and overcoming host defense mechanisms [14,15]. Oral delivery, transport across the BBB, stimulus-responsive release, and synergistic treatment approaches characterize the potential of nanotheranostics. The integration of diagnostic and therapeutic agents within nanomedicine allows the creation of single theranostic doses conjugated with biological ligands for targeted delivery [16].

**Fig. 1:**
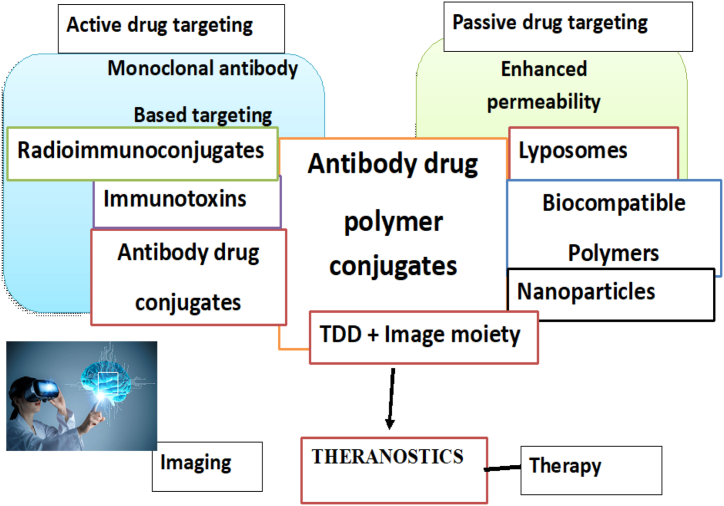
Schematic overview of theranostics in various cancers.

Within this domain, drug‒polymer conjugates, polymeric/magnetic theranostics, gold nanoparticles, SLNs, liposomes, micelles, carbon nanomaterials and dendrimers have emerged as key players [17]. Drug‒polymer conjugates offer covalent interactions for controlled drug release and targeted delivery, while polymeric/magnetic theranostics combine imaging and therapeutic functionalities for enhanced outcomes [Fig. 1]. With their unique properties, SLNs can serve as carriers for targeted therapy and imaging to treat diseases such as atherosclerosis and glioblastoma. Dendrimers, liposomes, and micelles, each with specific characteristics, contribute to the versatility of nanotheranostics. Gold nanoparticles and carbon nanomaterials, which leverage their distinctive properties, present promising avenues for treating multidrug-resistant tumours and advancing theranostic applications [[17], [18], [19]].

### 1.4 Biomimetics: nature as a blueprint for theranostic innovation

As we delve into the realm of cancer theranostics, the convergence of biomimetics with nanotechnology has emerged as a focal point for innovation. Biomimetics, or bioinspired design, draws inspiration from nature to develop technologies that emulate biological systems [20]. Nature, with its unparalleled efficiency and sophistication, provides a rich source of inspiration for overcoming challenges in cancer diagnosis and treatment [21].

The intricate biological processes within living organisms serve as blueprints for the development of nanotheranostic systems. The ability of nature to navigate complex physiological barriers, target specific tissues, and respond dynamically to environmental cues has inspired the design of nanocarriers capable of mimicking these functionalities [22,23]. Biomimetic nanomaterials, informed by the principles of natural selection, exhibit enhanced biocompatibility, reduced immunogenicity, and improved target specificity [24].

Biomimetic approaches in cancer theranostics are poised to revolutionize treatment strategies. By harnessing the inherent properties of biomolecules, such as proteins, peptides, and lipids, researchers aim to enhance the selectivity of theranostic agents for tumor cells while minimizing off-target effects [25,26]. For instance, the use of biomimetic nanoparticles, which model cell membranes, has shown promise for preventing invasion of the immune system, prolonging circulation time, and improving targeted delivery.

### 1.5 Significance of biomimetic nanotheranostics in cancer treatment

The significance of biomimetic nanotheranostics in cancer treatment lies in their ability to address critical challenges associated with conventional therapeutic modalities. Multidrug resistance, a pervasive obstacle in cancer treatment, often limits the effectiveness of chemotherapy [27]. Biomimetic strategies, inspired by the complexity of cellular membranes, aim to outsmart cancer cells by evading their defense mechanisms. Through the integration of biomimetic nanocarriers, researchers aspire to overcome multidrug resistance and enhance the therapeutic efficacy of anticancer drugs [28,29].

Biomimetic nanotheranostics also play an essential role in cancer immunotherapy, an evolving frontier in cancer therapy. By mimicking the body's immune response, biomimetic nanoparticles can stimulate and modulate immune cells to recognize and target cancer cells more effectively [30]. This personalized and targeted approach holds immense potential for improving overall outcomes of immunotherapy, minimizing side effects, and expanding its applicability across diverse cancer types [[31], [32], [33]].

**Fig. 2:**
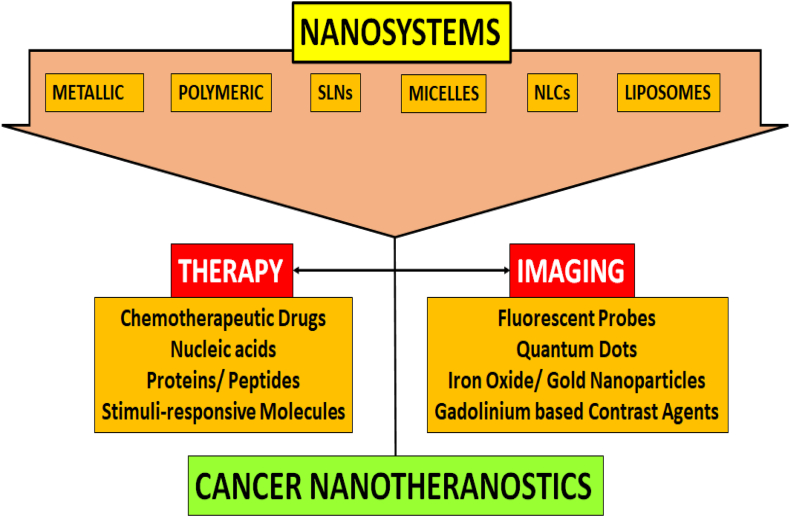
Nanotheranostics employing imaging and therapy techniques.

Moreover, biomimetic nanotheranostics have contributed to the field of photothermal therapy, where light-absorbing nanomaterials are employed to generate localized heat and selectively destroy cancer cells [Fig. 2]. The design principles inspired by nature enable the development of nanocarriers with enhanced photothermal conversion efficiency, enabling precise control over therapeutic outcomes [34].

### 1.6 Objectives of the comprehensive review

This comprehensive review aims to fulfil several objectives:

Survey of Biomimetic Strategies in Cancer Theranostics: We undertook an exhaustive exploration of biomimetic approaches, ranging from the design of nanocarriers to the integration of biomolecular components, to enhance the specificity and efficacy of cancer theranostics [35].

Addressing Multidrug Resistance: We scrutinize how biomimetic nanotheranostics contribute to overcoming multidrug resistance, a formidable challenge in cancer treatment, by leveraging nature-inspired design principles [36].

Advancements in Cancer Immunotherapy: This review explores the role of biomimetic nanotheranostics in advancing cancer immunotherapy, with a focus on personalized and targeted strategies that mimic the body's immune response [37].

Contributions to Photothermal Therapy: We explored the significance of biomimetic nanotheranostics in photothermal therapy, shedding light on their potential to enhance the precision and efficacy of this emerging therapeutic modality [38].

Applications in Personalized Medicine: This comprehensive review investigates how biomimetic nanotheranostics align with the principles of personalized medicine, contributing to tailored approaches based on individual genetic profiles and disease characteristics [39].

Through a detailed analysis of these objectives, we seek to provide a better understanding of the current landscape and forthcoming directions of biomimetic cancer theranostics. By critically examining the latest advancements and challenges in the field, we aim to guide researchers, clinicians, and practitioners toward innovative solutions that harness the potential of nature-inspired designs for improved cancer diagnosis and management [40].

In the subsequent sections of this review, we delve into specific biomimetic strategies employed in cancer theranostics, exploring their applications in targeted drug delivery, controlled release, and diagnostic imaging [41,42]. Additionally, we assessed the role of biomimetic nanomaterials in pretargeting techniques, cancer imaging and diagnosis and their implications for personalized and precision medicine. Through this exploration, we aspire to contribute to the ongoing dialogue in the scientific community, fostering a deeper understanding of the transformative impact that biomimetics can have on advancing cancer theranostics and key insights, as indicated in Table 1 [[43], [44], [45], [46], [47]].

**Table 1:** Key insights into cancer theranostics.

Focus	Key Points
Basics of Cancer Theranostics	➢ Coined term "theranostic" by John Funkhouser for targeted and personalized treatments. ➢ Integration of diagnostics and therapeutics for a comprehensive therapeutic paradigm. ➢ Evolution of traditional medicine to proactive, science-based approaches. ➢ Modern medicine's reliance on clinical signs, imaging, and genetic profiling for diagnosis and treatment.
Revolutionizing Personalized Medicine	➢ Theranostics' impact on targeted and individualized therapies. ➢ Global spending on ineffective treatments highlights the need for specific therapies. ➢ Cancer cells' uncontrolled growth and disruption of telomeres. ➢ Theranostics, especially with nanotechnology, operating in diagnosis, therapy, and response.
Nano-Theranostic Advancements	➢ Nanotheranostics bypass first-pass metabolism, achieve systemic circulation, and overcome host defense mechanisms. ➢ Advantages include oral delivery, blood‒brain barrier transport, stimulus-responsive release, and multimodal therapies. ➢ Diagnostic agents, like quantum dots and magnetic nanoparticles, play a crucial role. ➢ Theranostic nanoparticles like liposomes, dendrimers, and carbon nanotubes offer multifunctionality.
Drug‒Polymer Conjugates	➢ Covalent interactions for drug‒polymer conjugates with imaging agents. ➢ Stimulus-responsive release and targeted drug delivery for enhanced efficacy. ➢ Examples include N-(2-hydroxypropyl) methacrylamide (HPMA) and its applications of clinical trials.
Polymeric/Magnetic Theranostics	➢ Integration of polymeric and magnetic nanoparticles for enhanced imaging and therapeutic effects. ➢ pH-responsive polymeric nanoparticles for targeted tumor therapy. ➢ Advancement of iron oxide nanoparticles for MRI-guided photothermal therapy.
SLN Theranostic	➢ Solid lipid nanoparticles (SLNs) offer enhanced drug loading, controlled release, and imaging capabilities. ➢ Applications in atherosclerosis treatment and glioblastoma targeting.
Dendrimers	➢ Artificial nanomedicines with a cylindrical polymer structure for theranostic applications. ➢ Size range of 10–100 nm, three-dimensional geometrical design, and potential challenges.
Liposomes	➢ Bilayer vesicles with hydrophilic and hydrophobic properties for drug and diagnostic delivery. ➢ Advantages include biocompatibility, biodegradability, and potential drawbacks like low stability.
Micelles	➢ Colloidal structures with a hydrophobic core and hydrophilic shell for delivery. ➢ Advantages include small size, stable dispersion, and potential applications in cancer therapy.
Gold Nanoparticles	➢ Core sizes ranging from 1.5 to 10 nm for drug conjugation and targeted therapy. ➢ Unique properties, imaging capabilities, and potential for treating multidrug-resistant tumours.
Carbon Nanomaterials	➢ Exploration of carbon nanomaterials (nanocarbons) for theranostic applications. ➢ Categories include carbon dots, carbon nanotubes (CNTs), and two-dimensional (2D) graphene. ➢ Advantages of CNTs in tumor elimination and theranostic applications.

## 2 Theranostics in various cancers

### 2.1 Nanotheranostics for treatment of breast cancer

Most malignancies among women worldwide are breast cancer (BC). Between 70% and 80% of primary BCs can be treated according to published research. Advanced civilizations with distant metastases are nearly impossible to cure with conventional techniques. When technetium-99 m-methoxyisobutylisonitrile ([99mTc] Tc-MIBI) was used to diagnose dense breast lesions that were not apparent by mammography in the early 1990s, this marked the beginning of effective and impressive engagement in the direct detection of BC [48]. BC is now more convenient for patients because of the use of nuclear medicine imaging facilities. Specific and limited radiopharmaceuticals can be devised and produced based on the genetic categorization of BC, which can be divided into basal-like, luminal (A/B), HER2, and breast-like subtypes according to patient prognosis [49]. To treat severe pain in patients with metastatic disease, radiopharmaceuticals such as phosphorus-32 (32P), rhenium-186/188 (186/188Re), strontium-89 (89Sr), and samarium-153 (153Sm) can be used. The term "theranostic" refers to the use of radiolabelled ligands in conjunction with therapeutic radionuclides such as α or β emitters to achieve customized targeted therapy based on preliminary diagnostic procedures, as well as radiolabelled ligands in conjunction with diagnostic radionuclides such as gamma or positron (+) emitters [50].

Nanotheranostics refer to the integration of diagnostic and therapeutic functions into a single nanoscale platform. The advantages of both nanomedicine and theranostics include the ability to enable targeted delivery of drugs, imaging agents, and other therapeutic modalities to specific disease sites while also providing real-time monitoring of treatment response. Nanotheranostics hold great promise as a potential strategy to improve the diagnosis, treatment, and monitoring of these ailments [51].

NPs can be engineered to specifically target cancer cells or tumor-associated microenvironments. Functionalized nanoparticles can carry therapeutic agents such as chemical drugs, targeted therapies, or gene therapies directly to the tumor site, decreasing off-target effects and reducing systemic toxicity. Imaging and early detection: NPs can serve as contrast agents for various imaging techniques, including magnetic resonance imaging (MRI), positron emission tomography (PET), and near-infrared fluorescence imaging (NIRF). By incorporating imaging agents into theranostic platforms, it is possible to improve the sensitivity and specificity of BC detection, enabling early diagnosis and accurate tumor staging [52]. Nanotheranostics can provide real-time monitoring of treatment response by incorporating imaging agents that can visualize tumor regression, response to therapy, and the development of resistance. This allows clinicians to make informed decisions regarding treatment modifications and personalized medicine approaches [53].

In combination therapy, theranostic platforms can be designed to carry diverse therapeutic agents simultaneously, permitting combination therapy approaches. For example, nanoparticles can carry both chemotherapeutic drugs and targeted therapies to enhance the effectiveness of treatment and overcome drug resistance. Recent research on theranostic approaches for breast cancer has focused on improving the biocompatibility, stability, and targeting capabilities of nanomaterials, as well as integrating multiple functionalities within a single nanoplatform [54].

#### 2.1.1 BC diagnosis and treatment

Although distant metastases are typically thought of as secondary or late signs of BC, there is evidence to suggest that they can occasionally be early symptoms. For early detection, modern techniques such as digital breast cancer (BC) diagnosis have focused on early detection and personalized therapeutic approaches. Notable advancements include digital mammography (DM): This technique uses digital detectors to capture and analyse breast images, offering improved visualization and image manipulation compared to traditional film mammography (DM), MRI and molecular breast imaging (MBI) [55]. Patients are subjected to X-rays during mammography exams, and many false-positive results demand additional imaging or pathological testing. Radiolabelled molecules can be incorporated into m: MRI provides detailed images of the breast, which are particularly useful for high-risk individuals or when additional information is required after mammography. Molecular breast imaging (MBI) utilizes a radioactive tracer to detect breast abnormalities, especially in dense breast tissue where mammography may be less effective. Molecular imaging techniques such as single-photon emission computed tomography (SPECT/CT) and computed tomography (PET/CT) combined with functional imaging (SPECT) and anatomical imaging can increase the accuracy of cancer staging, response assessment, restaging, and recurrence detection for cancer management [56]. Moreover, PET/CT uses radiolabelled molecules to visualize metabolic activity in tumour cells, aiding in diagnosis, staging, and treatment monitoring. Therapeutic radiopharmaceuticals in nuclear medicine are noninvasive and have fewer adverse effects than therapeutic radiopharmaceuticals, which deliver targeted radiation to cancer cells. These treatments have fewer adverse effects than surgery; other conventional radiation procedures, including surgery; radiation treatment; chemotherapy; and endocrine (hormone) therapy. chemotherapy, or hormone therapy. These advances complement other established diagnostic tools, such as biopsy and histopathological analysis, and treatment modalities, such as targeted therapy, surgery, radiation therapy, hormone therapy and chemotherapy [57].

#### 2.1.2 Targeting HER2 receptors by therapy: BC

The HER2-HER3 dimer is the most remarkable dimer for achieving diagnostic and therapeutic goals. The activation of these receptors triggers complex signal transduction pathways that promote tumorigenic processes. HER2 is currently a vital oncogene in BC. Anti-HER2 therapy is often utilized in systemic therapies for HER2-positive patients. Since HER2 is a well-known factor whose amplification causes uncontrolled cell proliferation in BC, advanced approaches for BC detection and therapy have been developed. The most frequent procedure used to treat BC is the administration of the anti-HER2 monoclonal antibody "trastuzumab". An evaluation of the pretreatment physiology of trastuzumab would be important given that this therapeutic strategy imposes large costs on the patient and may still be ineffective [58].

Several efforts have been made to prepare PET derivatives for the diagnosis of metastatic lesions in BC patients because PET offers improved resolution and detection sensitivity. Cooper-64 (64Cu) is a positron-emitting radionuclide. Because of these properties, copper-64 is an effective PET radionuclide for high-quality detection. The final radiopharmaceutical can be transported since copper-64 has a longer half-life than other PET radionuclides. It is also suitable for radiolabelling substances with longer biological half-lives, including monoclonal antibodies. Six patients with primary or metastatic HER2-positive BC were treated clinically with [64Cu] Cu-trastuzumab. All patients were treated with an intravenous (i.v.) injection of 130 MBq [64Cu] Cu-trastuzumab, followed by diagnostic tests one, twenty-four, and 48 h later [59]. This study showed that the sensitivity of [64Cu] Cu-trastuzumab for the detection of brain metastases can be on par with that of MR imaging and even better than that of CT imaging under specific conditions. Overall, this clinical research suggested that the [64Cu] Cu-trastuzumab diagnostic approach is feasible, reproducible, and safe. This study's diagnostic scan was unable to detect trastuzumab therapy during surgery, and the sensitivity of the technique was sufficient [60].

#### 2.1.3 Breast cancer-theranostic radiopharmaceuticals: targeting GRPR

There are three known subtypes of bombesin receptors (BB1, BB2, and BB3). BB2, formerly known as GRPR, specifically binds to gastrin-releasing peptide (GRP) and is a promising option for directing and treating pancreatic cancer (PC). Bombesin, a 27-amino-acid peptide, shares the same functional C-terminal group as GRP. Additionally, GRP has been identified as a growth factor in both healthy and malignant cells [61]. BB2 is known to be overexpressed in diverse cancers, such as ovarian, pancreatic, lung, breast, and prostate cancers, as well as in CNS tumours (glioma, meningioma, and neuroblastoma). Preclinical studies involving breast cancer cell lines and animal research have shown encouraging results, with radiopharmaceuticals selectively accumulating in breast cancer [62].

Clinical trials using radiolabelled bombesin receptor (BnR) agonists have been conducted based on these promising outcomes. A recent clinical trial utilized a newly developed FAPI derivative called DOTA-SA. The FAPI was used to diagnose breast cancer in a 31-year-old female patient. Like FAPI-02 and FAPI-04, this novel FAPI is considered to serve as a diagnostic agent by radiolabelling it with gallium-68, lutetium-177 (177Lu), or actinium-225 (225Ac). The results of the trial revealed significant associations between the uptake of [68Ga]Ga-DOTA.SA. [18F] FDG and FAPI [63]. The patient's initial treatment involved the administration of 3.2 GBq of [177Lu] Lu-DOTASA. Posttreatment imaging was subsequently conducted 24 h after surgery. The absorbed dosage patterns observed in the lesions caused by [68Ga] Ga-DOTA.SA. FAPI and [177Lu] Lu-DOTA.SA. The FAPI was found to be similar. Although theranostic applications in nuclear medicine utilizing radionuclides such as gallium-68 and lutetium-177 have significantly progressed in recent years, especially with the improvement of [68Ga]Ga-PSMA-11 for the treatment of PC, advances in new drugs targeting BC have been relatively rare. Existing drugs for BC treatment are primarily designed to aid in diagnosis. Undeniably, the use of theranostic radiopharmaceuticals offers substantial advantages in nuclear medicine. This approach enables focused treatment planning and simultaneous evaluation of treatment response, providing numerous benefits [[64], [65], [66]].

### 2.2 Treatments for colon cancer

Colon cancer poses a considerable global health threat, with more than 1.8 million new cases reported annually. This disease affects the colon or large intestine and ranks as the third most prevalent cancer in both sexes worldwide. Despite improvements in diagnosis and management, colon cancer continues to contribute significantly to illness and death. However, the development of theranostic agents could transform cancer treatment, including for colon cancer treatment. This essay examines the concept of theranostics and their use in colon cancer treatment, along with potential future impacts and challenges [67].

Colon cancer is a malignant tumor that can develop in the lining of the colon. It typically begins as a small growth called a polyp, which can eventually become cancerous if left untreated. Current diagnostic methods for colon cancer include colonoscopy, faecal occult blood tests, and imaging studies of CT scans [68]. However, these traditional diagnostic methods have limitations, including the invasiveness of colonoscopy and lack of sensitivity and specificity of faecal occult blood tests and imaging studies.

New oncological approaches involving combinations of diagnostic and therapeutic capabilities of a single agent have emerged as promising treatment strategies. One potential theranostic agent for colon cancer is USPIO NPs with different coatings, which are currently under development for imaging and treatment purposes. Additionally, endogenous hydrogen sulfide has been used to activate the function of theranostic agents, enhancing the efficacy of colon cancer treatment. Moreover, fibroblast activation protein (FAP) is selectively expressed in the stromal fibroblasts of colon cancer tumours and has received significant consideration as a target for theranostic agents [69].

A diagnostic test is used to identify specific molecular targets in cancer, followed by the administration of a therapeutic agent that selectively targets those targets [70]. In the case of colon cancer, theranostics such as EGFR can be used to identify specific molecular targets in cancer cells, after which patients can be administered targeted therapies such as cetuximab or panitumumab. Compared with traditional cancer treatments, theranostics have several advantages, including increased efficacy, reduced toxicity, and improved patient outcomes. As a platform for theranostic nanomaterials in colon cancer (CC) treatment, USPIO NPs have recently attracted increasing interest. In addition to colon cancer, theranostic potential has also been examined for other types of cancers, such as breast, lung, liver, and prostate cancer [71].

The use of endogenous hydrogen sulfide to activate the diagnostic and therapeutic functions of smart theranostic agents is an effective strategy for reducing the rate of misdiagnosis and improving therapeutic efficacy in CC patients. Furthermore, recent studies have shown that neodymium-doped HAp NPs can serve as smart theranostic nanoplatforms for colorectal cancer treatment. Interestingly, PSMA expression in the tumor neovasculature of some solid tumours potentially prevents imaging but also offers potential opportunities for theranostic applications in cancers other than prostate cancer, including colon cancer. With continuing progress, theranostic agents have shown immense potential for the treatment of colon cancer [72].

This approach has gained significant attention in recent years because it offers personalized and targeted treatment options that can significantly improve CC patient outcomes. Various theranostic agents have been developed and explored for their efficacy in colon cancer treatment. Some promising theranostic agents for colon cancer include USPIO NPs, endogenous hydrogen sulfide-activated smart theranostic agents, and neodymium-doped HAp NPs [73]. Furthermore, FAP, which is selectively expressed in the stromal fibroblasts of colon cancer tumours, has also been identified as a valuable theranostic target. Along with other types of cancer, theranostic agents have emerged as promising agents for the diagnosis and treatment of CC. Research in the field of theranostics is ongoing, and potential avenues for future exploration include developing theranostic agents with enhanced specificity and efficacy for cancer cells, improving the delivery and distribution of theranostic agents within tumours and exploring synergistic agents. The use of endogenous hydrogen sulfide to activate diagnostic and therapeutic functions of smart theranostic agents has been shown to be an effective strategy for reducing the rate of misdiagnosis and improving treatment efficacy in patients with CC [74].

Various nanomaterials, such as Cu-based metal-organic frameworks and CuO, have been explored for designing H2S-responsive theranostic agents for colon cancer treatment. Additionally, neodymium-doped HAp NPs have demonstrated considerable promise as smart theranostic nanoplatforms for colorectal cancer management as well as for other cancers (breast, lung, and liver cancer) [75]. Another promising theranostic agent for colon cancer treatment is USPIO NPs, which have multiple applications with different coatings under development as new contrast and therapeutic agents. As the field of theranostic approaches continues to evolve, they hold immense potential for improving patient outcomes in CC. In particular, FAP has been identified as a promising theranostic target in CC [76]. The future implications of theranostic approaches in CC treatment are promising, with potential developments in personalized medicine and targeted therapies. However, there are also limitations and challenges associated with the use of theranostic agents, including the high cost of development and production, the need for specialized equipment and expertise, and ethical considerations such as the potential for genetic discrimination. As such, a multidisciplinary approach is necessary to address these challenges and ensure the safe and effective use of theranostic agents in CC treatment.

### 2.3 Theranostics for colorectal cancer

Despite substantial progress and improvements in treating colorectal cancer using targeted therapies, metastatic colorectal cancer remains an incurable condition. The emerging field of nanotheranostics offers a glimmer of hope and promises to improve the prognosis and progression of this disease. Globally, various innovative nanoparticles are being designed to revolutionize drug delivery in colorectal cancer, aiming for increased drug concentrations, reduced toxicity, and improved specificity. Many types of nanoparticles tailored for colorectal cancer treatment, such as polymeric nanospheres, micelle particles, metal-semiconductor nanoparticles, and gold nanoparticles, are under development, akin to their application in pancreatic and gastroesophageal cancers [77].

### 2.4 Pancreatic cancer theranostic efficacy

Before discovering the most effective targeted therapy for each mutation, tumours must be classified according to their genetic mutations, changes, or overexpression. Although a significant number of oncogenic driver changes linked to carcinogenesis have been identified in lung adenocarcinoma and BC, only a few targeted treatments have been licenced for these two tumours. Numerous new targets in gastrointestinal malignancies are being identified and will most likely be implicated in future theranostic initiatives [78].

As oncogenic drivers, MET and FGFR2 overexpression, in addition to Her2neu overexpression, are recognized as potential therapeutic targets in gastric cancer. In particular, STAT3, along with P53 and SMAD4, has emerged as a potential future target in pancreatic cancer treatment. Research indicates that iron oxide nanotheranostics hold great promise for treating pancreatic cancer [79]. The National Cancer Institute's Alliance for Nanotechnology in Cancer is pioneering a new project to create a multifunctional theranostic nanoparticle platform that integrates nanoparticles with imaging capabilities and receptor specificity, along with novel designs for tumor-targeted delivery under pancreatic tumor conditions. This rapid increase in iron oxide theranostic agents may improve the prognosis of pancreatic cancer patients over the next decade [80].

#### 2.4.1 New theranostic targets in PC

Theranostics, the integration of therapeutics and diagnostics, is an emerging field in medicine that aims to provide personalized and precise treatment options. Identifying new molecular targets is a crucial aspect of advancing theranostic approaches. Programmed cell death protein 1 (PD-1) and its ligand PD-L1, PD-1 and PD-L1, are immune checkpoint proteins that regulate the immune response [81]. Therapeutic antibodies targeting PD-1 or PD-L1, such as pembrolizumab and nivolumab, have been established for cancer immunotherapy. Additionally, PD-L1 expression can be used as a diagnostic marker to identify patients who may benefit from PD-1/PD-L1 blockade therapy [82].

### 2.5 Treatments for ovarian cancer

Gynecological malignancies contribute significantly to cancer-related fatalities in women, with ovarian cancer ranking seventh in terms of mortality. In the U.S., approximately 14,240 individuals succumb to ovarian cancer annually, and approximately 22,280 new cases are diagnosed. Metastatic disease, as in other cancer types, is the primary cause of death among ovarian cancer patients. Epithelial ovarian cancer, which constitutes nearly 90% of ovarian cancers, encompasses various subtypes, such as serous, endometrioid, clear cell, and mucinous [[83], [84], [85], [86]]. Most cases of epithelial ovarian cancer are diagnosed only after the cancer has extensively spread throughout the peritoneum, reaching an advanced stage. The 5-year survival rate for patients at this stage remains dismally low, between 30% and 40%, due to limited treatment options. The current standard therapy for high-grade epithelial ovarian cancer involves debulking surgery followed by chemotherapy using carboplatin and paclitaxel. However, the long-term outcomes associated with these drugs have been unsatisfactory. Debulking surgery serves as both a therapeutic intervention and for staging and diagnostic purposes [87]. The extent of residual disease postsurgery is an extrapolative factor for survival, and the complete absence of visible disease is associated with a lower recurrence rate. To mitigate the morbidity and mortality linked to ovarian cancer, there is an urgent need for additional targeted therapies.

#### 2.5.1 Ovarian cancer detection and molecular target therapy

MUC16/cancer antigen 125 (CA125) overexpression is a characteristic trait of high-grade serous ovarian cancer (HGSOC). These cancer cells express CA125, a mucin-type-*O*-linked glycoprotein, which is also soluble in bodily fluids. The serum CA125 concentration is a well-studied indicator of ovarian cancer and is assessed via immunoassays [88]. Clinically, CA125 levels are used for early finding, disease monitoring, initial outcome prediction, chemotherapy response assessment, and recurrence detection [89]. However, serum-based biomarkers such as CA125 levels are unable to reliably indicate the involved lymph nodes or site of recurrence, particularly in late-stage ovarian cancer (75% involvement). To address this issue, CA125-targeting PET probes, created by conjugating zirconium-89 or other imaging radionuclides with antibodies that bind to CA125, offer whole-body CA125 imaging and quantification. PET imaging with these probes can identify CA125-positive tumours as soon as 24 h after administration. Significant amounts of 89Zr-DFO-mAb-B43.13 have been detected in the lymph nodes of mouse models with lymph node involvement, indicating the potential of PET imaging for treating ovarian cancer [90].

#### 2.5.2 Folate receptor (FR)

A membrane glycoprotein anchored by glycosylphosphatidylinositol (GPI) that has a high affinity for folic acid and is overexpressed in more than 90% of HGSOC patients. Since there is little FR expression in healthy tissues, theranostic agents can be delivered to tumours only by FR900359. The internalization and sequestration of conjugates are the result of folic acid binding. Folate has been used to generate PET, SPECT, and fluorescence-based imaging agents in preclinical animals that produce highly resolved images of FR-positive tumours [91]. Numerous folate-derived conjugates, including the most frequently used radionuclides F-18 and Ga-68, have been created as PET imaging agents. Numerous FR-targeted therapeutics, including antibody–drug conjugates, have been developed because of FR overexpression in cancer [92].

Laparotomies of patients suspected of having ovarian cancer were performed with folate coupled with fluorescein isothiocyanate to view the tumours in real time. Fluorescence was observed intraoperatively in patients with FR-positive malignant tumours but not in those with FR-negative malignant tumours or benign tumours in that study, allowing for fluorescence-guided surgical removal of tumor deposits. By enabling better intraoperative staging and surgical resection, such medications may directly affect patient survival since tailored probes boost tumor detection during surgery. These findings were subsequently validated in another study in which an FR-targeting drug, EC17, was administered intravenously to ovarian cancer patients 2–3 h before surgery. The folate analogue EC17 was conjugated to 5-fluorescein isothiocyanate (FITC) [93].

Four transmembrane tyrosine kinase receptors from the EGF receptor (EGFR) or HER proto-oncogene family, which are involved in the etiology of many malignancies, such as ovarian cancer, are known to be significant therapeutic targets. Other cancers exhibit increased expression of the transmembrane glycoprotein HER2 (also known as Erbb2), which has a molecular weight of 185 kDa. One of most crucial biomarkers for guiding therapy is HER2 overexpression, which may give cancer cells a selective growth advantage. The effectiveness of anti-HER2 therapy has been validated and predicted using IHC and fluorescence in situ hybridization. HER2 overexpression has been observed to be highly variable in ovarian cancer [94].

The significant morbidity and mortality resulting from the intraperitoneal dissemination of ovarian cancer. An unmet medical need is the lack of low-toxicity treatments for i.p. disease. Although i.p. route chemotherapy has improved survival, it is a rare treatment and has potentially fatal side effects. By focusing radiation on cancer cells that express the target protein, targeted radiopharmaceutical therapy increases efficacy while lowering toxicity. These radiopharmaceutical treatments are frequently delivered using radionuclides that produce radiation. These radiotherapeutics have several benefits, such as a crossfire effect and an abscopal response, which are uncommon with conventional systemic medications. For the treatment of HER2-positive cancers and disseminated peritoneal disease, trastuzumab has been radiolabelled with and emits radionuclides [95].

EGFR, as a target in the spread of ovarian cancer through the intraperitoneal cavity, causes severe morbidity and lethality. There are few low-toxicity therapies for i.p. infection, which is an unmet medical need. Although i.p. route chemotherapy has increased survival, it is not a common treatment option and has life-threatening effects. Targeted radiopharmaceutical therapy directs radiation to tumours that express target proteins, increasing its efficacy while limiting its toxicity. These radiopharmaceutical therapies are frequently administered utilizing and emitting radionuclides. These radiotherapeutics have several advantages, including a crossfire effect and an abscopal response that are not commonly observed with traditional systemic medicines. Trastuzumab radiolabelled with and emitting radionuclides has been studied for the treatment of HER2-positive tumours and disseminated peritoneal illness [96].

Optimal tumor selectivity and quenching were achieved by coupling seven BPD conjugates with an EGFR-targeting cetuximab antibody (Cet-BPD). Through EGFR internalization and lysosomal transport, Cet-BPD conjugates were localized to lysosomes and subsequently destroyed. This process led to the intracellular release of BPD and dequenching, activating fluorescence emission and causing phototoxicity. Using a fluorescence microendoscope, the in vivo pharmacokinetics of the immunoconjugates were quantified, and metastatic burden reduction was monitored without the need for surgery, thereby reducing nonspecific phototoxicity. This approach shows potential for addressing metastatic disease [97].

#### 2.5.3 Ovarian cancer-targeting advances: new targets

Recently, a groundbreaking clinical trial involving ultrasound molecular imaging was conducted in patients with BC and ovarian lesions. This trial utilized a clinical-grade microbubble contrast agent called MBKDR, which specifically targets the kinase insert domain receptor (KDR). KDR plays a crucial role in the neoangiogenesis process in cancer and is particularly important for the growth and spread of ovarian cancer. The primary objective of this phase I study was to evaluate the safety of MBKDR and quantify KDR expression using the gold-standard immunohistochemistry (IHC) technique. Patients with localized ovarian or breast lesions received an intravenous injection of MBKDR, followed by ultrasonography, molecular imaging, and surgical removal of the lesions. The resected tissues were then stained with CD31 and KDR antibodies for analysis [98]. The findings from this trial indicated that the MBKDR was well tolerated by patients, and there was a strong correlation between the KDR expression observed via IHC and that detected via ultrasound imaging in 85% of malignant ovarian lesions. Additionally, a robust KDR-targeted signal was detected in 77% of the malignant ovarian tumours, while it was absent in 78% of the benign lesions.

In investigations involving near-infrared photoimmunotherapy (NIR-PIT), antibodies are commonly employed as targeting moieties. However, Harada et al. developed and tested a non-antibody-derived NIR-PIT agent for disseminated ovarian cancer. They used galactosyl serum albumin (GSA), which consists of galactose molecules attached to albumin via carboxyl groups. GSA can bind to the beta-D-galactose receptor, a surface lectin that is more highly expressed in various malignancies, in addition to ovarian cancer. Upon binding to its ligands, the beta-D-galactose receptor is rapidly internalized [99]. To evaluate the efficacy of the agent, they utilized SHIN3 cells, which exhibit high expression of the galactose receptor and cause diffuse peritoneal dispersion. This study demonstrated that the selective accumulation of the GSA-IR700 probe in tumours and repeated NIR-PIT treatments enhanced therapeutic effectiveness by allowing deeper delivery of GSA-IR700 into tumour nodules. These findings illustrated the potential of GSA-IR700 for targeted delivery to tumours.

Enzymatically triggered fluorescence probes have been investigated for use in visualizing ovarian cancer metastases, and galactosidase enzymatic activity was found to be elevated in approximately 50% of primary ovarian tumours compared to that in normal ovaries. Asanuma et al. established a fluorescent probe called HMRef-Gal, which can be administered intraperitoneally and enables the visualization of metastases as small as 1 mm in diameter within the peritoneal cavity. Recently, a topically sprayable and activatable fluorescent probe named SPiDER-Gal was developed to eliminate the need for intravenous injection before surgery. SPiDER-Gal attaches to intracellular proteins after activation by the galactosidase enzyme, allowing it to remain within cells. The efficacy of SPiDER-Gal was evaluated in vitro in cancer cell lines and *ex vivo* in tumor tissues. In a mouse model, compared with Glu-HMRG, a probe stimulated by glutamyl transpeptidase, SPiDER-Gal, demonstrated high sensitivity in detecting ovarian cancer metastases in the peritoneum. SPiDER-Gal exhibited superior signal retention and better visualization of the tumor margin than did Glu-HMRG. Moreover, SPiDER-Gal achieved a high target-to-background ratio due to significant amplification within the tumor, and signals remained detectable for up to 60 min after activation. These findings suggested that SPiDER-Gal-targeted probes could be used for laparotomic and endoscopic identification of primary tumours and metastases [100].

### 2.6 Theranostics in the treatment of cervical cancer

Cervical cancer is a global health concern, accounting for 8% of cancer-related deaths, with an estimated 500,000 new cases and 250,000 deaths annually. Screening methods such as cytology and high-risk HPV DNA testing have helped reduce disease prevalence and mortality [101]. However, a lack of healthcare access and living in underdeveloped areas continue to pose significant risks for women. For those with metastatic or inoperable recurrent disease, treatment options are limited. Surgical intervention is viable for early-stage disease, while chemo-radiotherapy can address locally progressed disease [[102], [103], [104]]. Palliative platinum-based chemotherapy typically results in a median overall survival of 8–12 months. Thus, improved knowledge of the molecular mechanisms underlying recurrent/metastatic cervical cancer and the development of efficient therapeutic strategies are urgently needed [105].

Membrane-spanning proteins called receptor tyrosine kinases (RTKs) have intracellular kinase activity that is controlled by ligands 5. Cancer development and progression are causally related to RTK deregulation caused by RTK overexpression, mutation, amplification, or autocrine activation. Our research focused on RTK modifications in the molecular pathogenesis of cervical cancer, and we observed significant changes in the KIT, PDGFRA, VEGFR2, and EGFR families of receptors [106]. Since various oncogenic changes have been found to potentially predict the response to anti-RTK and anti-signalling protein medicines, RTKs and intracellular signalling pathways represent intriguing targets for cervical cancer therapy [107].

HER receptors are members of RTK family I, along with HER2, HER3, HER4, and EGFR. These RTKs can recognize 13 growth factors, such as EGF, that have structural similarities. However, no soluble ligand for HER2 has yet been discovered. Human breast, ovarian, stomach, and brain malignancies have all been linked to the origin or progression of HER family receptor amplification and coexpression [108]. Various cancers have also been shown to exhibit HER2 abnormalities. HER2 aberrations are present in between 1% and 37% of tumours, including germ cell, glioma, and lung, ovary, and salivary duct [109].

Although HER receptor expression is elevated in cervical cancer, its prognostic and therapeutic usefulness have yet to be determined. Patients with cervical cancer are being treated with RTK inhibitors in several clinical studies; however, the anticipated advantages have not been observed, primarily because cases were not preselected for molecular changes in targets of interest. Therefore, except for bevacizumab, no other targeted treatments, including those intended to target RTKs, have received approval for the treatment of cervical cancer [110].

#### 2.6.1 The role of HERRs in cervical cancer

HER receptors exhibit altered mRNA expression, gene copy counts, and mutation rates, with HER2 exhibiting the most frequent changes (13% - 25/190). Tyrosine kinase inhibitors may be used to target EGFR and HER2 because they both cause mutations in several tyrosine kinase phosphorylation sites. Additionally, patients with HER2 mRNA upregulation and protein overexpression appeared to be the only patients with HER2 gene amplification, with 3% of patients (6/190) exhibiting HER2 gene amplification [111].

#### 2.6.2 Targetable proteins on cervical cancer cell lines include HER receptors

According to the Cancer Cell Lines Project Database (COSMIC), there are no RTK genetic alterations in the HeLa, SiHa, Caski, or C-33A cervical cancer cell lines. Intriguingly, it was discovered that only the HER family of receptors was activated when we used a phospho-RTK array to determine the basal levels of RTK activity. Western blotting was used to evaluate the basal levels of EGFR, HER4, and HER2 activation in the SiHa, HeLa, and CaSki cell lines as well as in the C-33A and CaSki cell lines. All the cell lines exhibited phosphorylated EGFR in response to EGF stimulation, whereas the SiHa, HeLa, and CaSki cell lines exhibited phosphorylated HER2 [112]. The surface localization of HERRs was verified by immunofluorescence since plasma membrane localization is crucial for determining treatment success.

To assess the potential for precise and effective targeting of the HER family of receptors in cervical cancer treatment, the sensitivity of various cell lines to several pan-RTK inhibitors was evaluated. AST1306 and lapatinib displayed significantly lower IC50 values than other inhibitors, which were intended to primarily target EGFR (erlotinib) or multiple RTKs (cediranib, sunitinib, and imatinib). Subsequently, researchers explored how each drug influenced the blockage of HER signalling pathways to gain insights into the cytotoxic mechanisms of inhibitors [113]. The key factor contributing to the maximum cytotoxic effect of AST1306 and lapatinib appears to be their ability to simultaneously block the phosphorylation of HER2, HER4, EGFR, and other proteins, such as ERK and AKT.

Lapatinib combined with AKT (MK2206) and MAPK (selumetinib) pathway inhibitors was combined in C-33A and SiHa cell lines, which have different responses to and dependencies on these pathways, to determine whether simultaneous inhibition of RTKs and intracellular pathways is necessary for an effective response to these inhibitors. The IC50 value of lapatinib may be reduced by either medication; however, the impact was greater in less sensitive and EGF-dependent SiHa cell lines. Furthermore, the response of SiHa cells to lapatinib was nearly doubled by the combined inhibition of EGFR (with lapatinib) and the AKT pathway (with MK2206) [114].

In vitro and in vivo, the inhibition of HER receptors can decrease the aggressiveness of cervical cancer cells. We selected the extremely sensitive cell line C-33A and the comparatively less sensitive cell line SiHa to examine the biological effects of HER inhibitors on cell survival, migration, and proliferation both in vitro and in vivo. Due to their potent cytotoxic effects on both cell lines, lapatinib and, markedly, AST1306, even at lower dosages, were shown to be more effective than erlotinib over time in a viability assay. PARP cleavage assessment revealed that the effectiveness of AST1306 was also a result of its improved capacity to induce apoptosis [115].

In line with the in vitro tests, lapatinib and AST1306 treatment decreased the growth of tumours created by injecting C-33A cells but not SiHa cells, even though both inhibitors are antiangiogenic agents for tumours created from SiHa cells [116]. The results of a histological study showed that both medications were biologically active in preventing tumor development in both models [117]. Large necrotic regions formed in the treated tumours due to a lack of cellular viability and integrity, as well as a decrease in the expression of relevant HER targets and the number/intensity of Ki-67-positive cells stained. Due to the limited experimental window in the CAM assay, cell death and suppression of proliferation were observed in vivo even when the tumor volume was not reduced [118].

### 2.7 Gastric cancer treatments

Although predictive biomarkers frequently used in clinical practice fall under the definition of theranostics, this definition still applies to many other facades for managing malignant disease. Theranostic nanomedicine is being examined from both present and near-future perspectives as a means of obtaining more refined personal medicine that better caters to the needs of each patient. This new idea requires further investigation of nanoparticles (NPs), artificial particles with sizes between tens and hundreds of nanometres that have grown in popularity over the past 10 years due to their effectiveness in delivering drugs with minimal systemic toxicity, such as albumin-bound paclitaxel nanoparticles (Abraxane) [119]. Some of these NP experimental models, which are referred to as "smart" NPs, have no clinical applications outside of animal models yet, but the desired outcomes are easily implementable in contemporary practice. When the microenvironment changes, "activatable" NPs can act to implement therapeutic or diagnostic mechanisms. As a result, NPs might be selective for a particular tumor environment, such as an acidic pH caused by tumor hypoxia and the concomitant formation of lactic acid, before releasing their payload. Other types of NPs can be activated by irradiation with a specific wavelength of light or protease and are known to be upregulated by tumours. This opens the door for advances in multifunctional NPs that have simultaneous diagnostic and therapeutic applications. The desire for less invasive and more targeted therapy options is increasing, and this will undoubtedly result in continued advancements in theranostic NPs and their impact on clinical cancer in the years to come. Our present goal is to obtain NPs that can recognize cancerous clones and treat them with the best possible drug delivery. The creation of "nanobots," which are referred to as "artificial cells," which continuously circulate in the host's system and activate theranostics at the first illness stage, would be a more far-reaching goal [120].

#### 2.7.1 Theranostics for gastrointestinal and pancreatic neuroendocrine tumours

Different neuroendocrine cells found throughout the human body give rise to neuroendocrine tumours; these cells have granules that secrete amines and peptides. The broncopulmonary and gastrointestinal tracts are the most common locations for these tumours. NETs often have a good prognosis, with a five-year survival rate of approximately 80%. The therapeutic application of radiolabelled ligands for imaging and therapy in these tumours is made possible by the distinctive properties of NETs, which include the presence of peptide receptors and transporters at the cell membrane and neuroamine absorption pathways [121]. Due to the prevalence of somatostatin receptor expression in NETs, this tumor type has become the best candidate for somatostatin receptor therapy. The value of somatostatin targeting as a diagnostic and therapeutic tool for NET tumours has increased significantly in GI theranostics [122]. Somatostatin receptor imaging relies on the use of whole-body techniques such as positron emission tomography (PET) or single-photon emission computed tomography (SPECT) (scintigraphy); varieties of tracers are included in panels used for imaging with varying degrees of sensitivity and specificity. For patients with metastasized and unresectable NETs, combining somatostatin analogues with therapeutic beta emitters (lutetium-177 and yttrium-90) is regarded as an effective therapeutic alternative in addition to its imaging benefit. Somatostatin receptors, common imaging and therapeutic targets in NETs, are thought to be key to the initial success of theranostic treatment for GI tumours and various strategies, as depicted in Table 2 [123].

**Table 2:** Biomimetic theranostic strategies for various cancers.

Types	Biomimetic Nanotheranostic Strategy	**Drug**	Diagnostic Modality	Key Findings
Breast Cancer	Liposomal Nanocarriers Mimicking Cell Membranes	Doxorubicin, Paclitaxel	Near-Infrared Imaging, MRI	Enhanced drug delivery, Reduced side effects
Lung Cancer	Targeted Nanoparticles with Peptide Ligands	Erlotinib, Cisplatin	PET-CT Imaging	Improved Targeting, Early Detection
Prostate Cancer	Polymeric Micelles for Controlled Drug Release	Docetaxel, Cabazitaxel	MRI, Ultrasound Imaging	Prolonged Drug Release, Enhanced Imaging
Colorectal Cancer	Dendrimer-based Theranostic Nanoparticles	5-Fluorouracil, Oxaliplatin	CT Imaging	Overcoming Drug Resistance, Targeted Delivery
Ovarian Cancer	Gold Nanoparticles with Targeting Ligands	Paclitaxel, Cisplatin	Optical Imaging, Ultrasound	Improved Therapeutic Efficacy, Minimized Toxicity
Pancreatic Cancer	Biomimetic Liposomes with Immunotherapy Components	Gemcitabine, Nab-paclitaxel	PET Imaging	Synergistic Immunotherapy, Improved Patient Outcomes
Skin Cancer	Nanoscale Lipid Nanoparticles for Topical Delivery	Imiquimod, 5-Fluorouracil	Dermoscopic Imaging	Enhanced Topical Delivery, Precise Lesion Targeting

#### 2.7.2 Her2neu in gastric cancer and KRAS in colon cancer

Only 2 molecular mutations in GI cancers have been identified as therapeutic targets for prediction. KRAS is a negative predictor of metastatic colon cancer, whereas Her2neu is a positive predictor of advanced gastric cancer. A monoclonal antibody against HER2 (ERBB2) is known to be a good predictor of response because of the overexpression of Her2neu in advanced gastric and gastroesophageal junction tumours. In the TOGA study, individuals with Her2neu overexpression who received trastuzumab combined with chemotherapy as first-line therapy had an overall survival of 13.8 mo, compared to the one-year overall survival in the population who did not receive trastuzumab [124,125]. For the treatment of metastatic colon cancer with cetuximab, an anti-EGFR KRAS mutation is a poor prognostic indicator. Patients with KRAS mutations do not benefit from cetuximab as much as do those with wild-type KRAS; bevacizumab is used more frequently to treat these patients [126].

#### 2.7.3 Future perspectives of theranostics in GI cancers

Some of these nanoplatforms target metaplasia, which is the precursor to both gastric and esophageal adenocarcinoma. Theranostics for treating gastroesophageal cancer are being explored using polypeptide nanoparticles, magnetic iron nanoparticles, and triblock copolymer nanoparticles. None of these NPs are used in clinical trials or for sale [127,128].

### 2.8 Pancreatic cancer

The resistance of cancer cells to chemotherapy and extensive formation of stroma around tumours make this malignancy difficult to treat. The adoption of nab-paclitaxel as a treatment option for metastatic pancreatic cancer is one specific example of how nanosized cytotoxic drugs have demonstrated greater pharmacological efficacy. In pancreatic cancer, various kinds of nanoparticles are being studied; iron oxide is one of the subtypes with the most encouraging outcomes in this form of disease [129]. Numerous studies have examined various methods of using iron oxide nanoparticles as theranostic agents, such as chemical delivery vehicles or gold-coated iron oxide nanoparticles. All these trials demonstrated the promising potential of iron oxide nanotheranostics for treating pancreatic cancer. Additional research should be performed to determine the parameters and specific mechanisms of action of these nanoparticles [130].

The National Cancer Institute Alliance for Nanotechnology in Cancer has started a new project to create a multifunctional theranostic nanoparticle platform that combines novel designs for pancreatic cancer drug delivery that target tumours with imaging capabilities and receptor specificity. Within the next ten years, early detection and treatment of pancreatic cancer using iron oxide theranostics may improve patient prognosis [131].

#### 2.8.1 New molecular targets in pancreatic cancer

Prior to identifying suitable targeted therapy for each mutation, the stratification of cancers based on their molecular mutations, alterations, or overexpression remains a pivotal step in personalized medicine. Although a considerable number of oncogenic driver mutations contribute to the carcinogenesis of lung adenocarcinoma and BC, only a small fraction of targeted therapies are currently approved for these malignancies [132]. Multiple new targets for gastrointestinal malignancies are continually being discovered and will likely play crucial roles in future theranostic efforts. Given their roles as oncogenic drivers, MET and FGFR2 overexpression are considered significant therapeutic targets in gastric cancer, along with Her2neu overexpression. Furthermore, STAT3, along with P53 and SMAD4, has been identified as a prospective target for pancreatic cancer treatment. With these targets and several others in the arsenal, the development of novel theranostic treatment modalities is expected to be greatly facilitated [[133], [134], [135], [136]].

### 2.9 Nanotheranostics in the treatment of prostate cancer

Conventional single ligand-based targeting nanocarriers used in tumor therapy lack the ability to effectively deliver and target two distinct surface receptors overexpressed in tumours. As a solution, an active nanoparticle delivery approach involving fucoidan and hyaluronic acid is under exploration to enhance therapeutic effectiveness. This system (IOs) is composed of epigallocatechin gallate (EGCG), poly(D,L-lactide-co-glycoside; PLGA), and stable iron oxide nanoparticles. The latter allows for targeted molecular imaging of prostate cancers. This strategy may demonstrate the benefits of medication administration, which enhances cell growth suppression by causing apoptosis [137]. In addition, compared to systemic combination therapy, the enhanced targeting of nanotheranostics greatly reduces the growth of orthotopic prostate cancers and more precisely targeted tumours [138].

Prostate cancer cells can disseminate to other parts of the body through bone marrow endothelial cells, which have increased levels of membrane-associated CD44. CD44^+^ prostate cancer cells that resemble stem cells invade and disseminate in vitro and in vivo. The CD44 molecules expressed by cancer cells alter the signalling pathways that promote malignant cell invasion and migration, leading to the initiation of metastatic tumor cell inflammation by interacting with HA-rich microenvironments [139]. In HA, a linear glycosaminoglycan with (14) interglycosidic linkages and N-acetyl-D-glucosamine and D-glucuronic acid disaccharide units alternate. Furthermore, HA can target cancer cells in nanoparticle (NP) delivery systems and increase cellular NP uptake. Poly(D,L-lactide-co-glycoside; PLGA) is commonly employed in NPs used in biomedical applications due to its high biocompatibility [140]. However, intravenously administered PLGA NPs are quickly eliminated from the blood by macrophages, similar to other traditional colloidal carriers. A water-soluble polymer called PEG can be used to modify gelatin to greatly reduce the cytotoxicity of the cancer drug doxorubicin and increase the stability of NPs. For instance, PEGylated PLGA NPs were used to delay opsonin binding, which slows the rapid uptake of NPs by the reticuloendothelial system [141].

### 2.10 Nanotheranostics in the treatment of skin cancer

UV radiation has two impacts on uncontrolled cell growth and keratinocyte death and is a major contributor to the progression of skin photoaging. Skin cancers (NMSCs) are classified into two types: melanoma and nonmelanoma. NMSC (MCC) is classified into three types: basal cell carcinoma (BCC), squamous cell carcinoma (SCC), and Merkel cell carcinoma [142]. Chemotherapy, adjuvant immunotherapy, and targeted therapy are advised after metastasis has occurred. Unfortunately, it is impossible to disregard the potential side effects of chemotherapy, such as typical cell damage, limited absorption, and tumor drug resistance.

Certain nanomaterials can accumulate at primary tumor sites, at lymph node metastases, or at distant metastases, enabling targeted imaging and powerful anticancer effects [143]. Functionalized nanoparticles have several advantages over conventional nanomedicine, such as improved pharmacokinetics, longer blood circulation times, and increased therapeutic effectiveness and delivery. Currently, two methods for altering nanoparticles are chemical and biofunctionalization. Additionally, these cutting-edge nanotechnologies help stabilize anticancer medications, enhancing bioavailability and controlled release.

#### 2.10.1 Functionalized nanomaterials for skin cancer treatment

Functionalization approaches using nanocarriers for drug administration or imaging agents have attracted the interest of researchers because of their ability to selectively target diseased organs and cells while sparing healthy tissues. Several functionalization processes have been used to modify and functionalize the surface of nanoparticles for application in cancer theranostics [144]. After intravenous administration, reticuloendothelial system (RES) macrophages, which are frequently found in the liver and spleen, opsonize and remove conventional nanoparticles from the bloodstream. The two primary methods of surface modification are chemical functionalization and biofunctionalization. Chemical methods such as amide coupling, click reactions, thiol coupling, and PEGylation have been used to achieve chemical functionalization [145].

#### 2.10.2 Functionalized liposomes

It is a phospholipid bilayer membrane that is expected to be a nontoxic and biodegradable encapsulating agent. Because of their great utility as a substitute for other therapeutic agents in delivering therapeutic components to the appropriate area, they have been employed to expand the therapeutic profile of anticancer medications while minimizing the incidence of unwanted effects. Nanocarriers aggregate inside tumor cells by passive targeting, which exploits the improved permeability and retention effect. Daunorubicin, doxorubicin, paclitaxel, and vincristine are only a few of the passively targeted liposomal medications for cancer therapy that have received FDA approval. The ability of RES to quickly remove liposomes is a significant obstacle. Poly(ethylene glycol) (PEG) conjugation to a liposomal membrane can prevent RES from absorbing lipids through the liposomal membrane. Compared to those of SA-NPs and salinomycin, the results of these studies showed that CD20-SA-NPs (salinomycin 5 mg kg1 d1, administered intravenously for 60 days) were more effective at preventing the formation of melanomas in mice harboring xenografts [146].

#### 2.10.3 Functionalized metal nanoparticles

The focus has shifted to the adoption of green alternatives as a method of overcoming the challenges they create due to the appearance of harmful side effects and the increased cost of processing processes used in the fabrication of silver nanoparticles (AgNPs). Because of their photostability and lack of toxicity, nanomaterials are being used to replace organic dyes. Noble metal nanoparticles, particularly gold and silver, are desirable due to their distinctive optoelectronic capabilities, which depend on their size and form. Noble metals are covered in inorganic/organic compounds, which renders nanoparticles less toxic and more biocompatible [[147], [148]].

#### 2.10.4 Functionalized polymeric nanoparticles

Polymeric nanoparticles were created to mitigate the loss and premature degradation of the medications they transport, which commonly occurs after enzymatic deactivation. They have the potential to increase drug accumulation in targeted body regions, reduce adverse effects, and enhance medication bioavailability. The inclusion of amphiphilic polymers in anticancer drug formulations significantly altered the release profiles of free drugs. Various types of polymer nanoparticles can be produced depending on the polymer's properties and intended applications [149].

Previous data showed that terpineol increases anticancer activity when integrated into PMMA/-terpineol NPs, as tested in melanoma-derived tumor cell lines. Moreover, no toxicity was observed in human macrophages or MRC-5 human fibroblasts, indicating that this formulation may be very successful at minimizing the side effects of many antineoplastic drugs when administered in their free form [150]. To target the vitamin D receptor (VDR) and encourage cell internalization, vitamin D3-functionalized lipid‒polymer hybrid nanoparticles have also been developed. Vitamin D3 was covalently bound to DSPE-PEG-2000 in these particles. According to these results, HNP-VD is a great method for creating customized melanoma treatment plans and delivering therapeutic compounds to additional cells that exhibit nuclear vitamin D receptors [151].

#### 2.10.5 Functionalized carbon nanotubes

CNTs have recently become increasingly significant due to their biocompatibility and capacity to transport large amounts of drugs and biomolecules. To target tumours while maintaining their NIR absorbance, maintain a stable dispersion for biocompatibility, and create SWCNT-based PTT materials, modifications to SWCNTs are needed. Under physiological circumstances, however, the NIR absorption of noncovalent or covalently functionalized SWCNTs may be swiftly reversed or diminished [152]. Antibody-conjugated gel-coated SWCNTs with stable characteristics and strong NIR absorption signals were created to overcome these restrictions. Using the PTT approach, researchers have proven that antibody-conjugated gel-coated SWCNTs are effective at locating and eliminating cancer cells. This inventive method for conjugating antibodies to SWCNTs will lay the groundwork for SWCNT-based platforms for the development of NIR photothermal agents in the near-infrared region [153].

### 2.11 Recent approaches for designing cancer theranostics

NPs exhibit an antigenic surface that is very comparable to that of cancer cells and serve as the source of NPs according to studies on functionalized NPs encased in cancer cell membranes. These NPs made it possible for immunological adjuvants and membrane-bound tumor-associated antigens to effectively reach cancer cells and stimulate an immune response. Mesoporous silica NPs hold great promise for theranostic uses. These agents are effective at treating a variety of malignant diseases in preclinical models and can be used in an extensive range of formulations [154]. Oral cancer has a low likelihood of survival and is difficult to treat. According to previous studies, reversing pingyangmycin and carboplatin medication resistance in oral cancer is possible when GST is properly inhibited by NPs, considerably improving the prognosis of the disease. The development of oxidative stress, which can have potentially fatal effects, is one of the factors to consider when utilizing NPs [155].

### 2.12 Magnetic resonance imaging

MRI can be used in conjunction with other forms of therapy to provide image-guided therapy for better therapeutic outcomes and the ability to target malignancies. In one study, an o-nitro-benzyl ester lipid was combined with a Gd-DTPA contrast agent to create a multifunctional Gd-DTPA-ONB lipid. To achieve maximum sensitivity without lowering the pace of drug encapsulation, MRI monitoring can be combined with dual trigger release functionality. It can be activated by both pH-triggered hydrolysis and light therapy [156].

#### 2.12.1 Immunotherapy

Cancer immunotherapy seeks to enhance the immune system's antitumour response and has benefits over chemotherapy, including fewer side effects that are not intended to occur. Advanced malignancies must be treated with T-cell checkpoint inhibitors [157]. Because each patient's immune response is unique, immunotherapy needs to be customized. Pharmaceuticals, such as monoclonal antibodies, immunological checkpoints, cell treatments, and vaccines, are all types of cancer immunotherapy. PD-1 as well as other immunotherapy medications, such as antibody‒drug conjugates and other therapies, including chemotherapy and radiation therapy, work together to cause programmed cell death [158].

#### 2.12.2 Photothermal and photodynamic therapy

Due to the advantages of their properties and theranostic compatibility, research on gold NPs has considerably increased in recent years. Due to their stability, improved solubility, functionality, biocompatibility, and capacity to target cancer cells, they have been broadly used in cancer therapeutics, including light imaging and PTT. In one study, hyaluronic acid, polyethene glycol, and adipic dihydrazide were used to functionalize AuNPs. A chemical approach was used to load the antitumour medication into the NPs. The outcome demonstrated that the NPs have greatly improved antitumour capabilities at high dosages while having very minimal toxicity towards cells [159]. The low penetration depth of PPTs is one of their drawbacks since it may result in inadequate removal of cancer cells, which could result in tumor recurrence and metastasis to other organs. It is possible to fix this flaw by combining PTT with other therapies [160].

#### 2.12.3 Molecular imaging

The detection of inflammatory cellular and molecular mechanisms in CVD highlights the enormous potential of molecular imaging. To identify vascular inflammation via molecular imaging, NPs have been examined as contrast agents. Microbubble technology may hold great promise for cancer detection [161].

### 2.13 Recent advances in cancer theranostic trials

Progress has been made in theranostic nuclear oncology over the span of 75 years. Currently, prospective randomized controlled multicenter clinical trials pave the way forward, revealing brightly and illuminating the path. By utilizing SPECT or PET/CT, cancer biomarkers can be quantified, allowing for the identification of tumoricidal radiation absorption doses. This enables the use of theranostic beta- or alpha-emitting radionuclide pairs connected to the same targeted molecule for the treatment of advanced metastatic cancer. Furthermore, groundbreaking advancements in immunological and molecular medications are revolutionizing the field of oncology [162]. Currently, multicenter randomized controlled trials are underway to assess the efficacy and safety of treatments that involve immune checkpoint blockade and CAR-T-cell therapy. Radioiodine therapy has long been considered the gold standard in theranostic nuclear oncology for well-differentiated thyroid cancer, but controlled prospective clinical trials for this treatment are lacking. To enhance outcomes and minimize myelotoxicity, experts recommend employing a more sophisticated radionuclide technique that combines PET/CT scans of iodine-124 with precise lesion and critical organ dosimetry [163].

One major hurdle in the advancement of iodine-131 RIT (radioimmunotherapy) for indolent non-Hodgkin lymphoma (NHL) lies in the failure of nuclear medicine professionals to effectively collaborate with medical hemato-oncologists. Issues related to logistics, politics, regulatory concerns, radiation safety, and reimbursement have hindered the integration of the iodine-131 RIT into mainstream clinical hemato-oncological practices. Despite the superior efficacy and improved safety profile of Bexxar, a medicine compared to conventional R–CHOP chemotherapy for NHL, it was withdrawn from the market in 2015 [163]. Notably, even with the support of a large pharmaceutical company's clinical trial resources, the lack of collaboration hampered its progress. Encouragingly, active preclinical research has unveiled new molecular targets that hold great promise for clinical application and theranostic approaches. Theranostics, as a concept, entails the fusion of diagnostics and therapeutics into a unified approach. By leveraging diagnostic tools to identify specific targets or biomarkers, followed by tailored targeted therapies for individual patients, theranostics represents a transformative approach in the field of medicine [164,165].

## 3 Future aspects of theranostic

Theranostics has emerged as a successful approach for treating diverse cancers, particularly prostate cancer and neuroendocrine tumours (NETs). The NETTER-1 trial provided evidence of the efficacy of NET therapy using radiolabelled analogues of somatostatin. Consequently, 177Lu-DOTATATE received approval in the U.S. and later in Europe. Concurrently, 68Ga-labelled somatostatin analogues for NET imaging and patient selection for peptide receptor radionuclide treatment (PRRT) were also approved. A more recent theranostic target for prostate cancer is PSMA [166]. Randomized phase III registration trials are currently enrolling patients to demonstrate the superiority of 177Lu-labelled PSMA-617 over current treatments for metastatic castration-resistant prostate cancer (mCRPC). Various PSMA-targeting imaging agents are undergoing phase I-III investigations to obtain approval as state-of-the-art diagnostic tools. These two theranostic approaches, along with the use of 223Ra to treat osseous prostate cancer metastases, have unequivocally shown the utility of radionuclide therapy not only in treating thyroid cancer but also in treating other solid cancers [167]. In addition to its palliative benefits, radionuclide treatment is now recognized to improve overall and progression-free survival. While new theranostic targets are currently being studied, it is crucial to optimize radiopharmaceutical doses and schedules, explore combination therapies, and treat tumours at early stages [168].

Promising preclinical cancer advancements, with a focus on immunology, hold the potential for identifying novel molecular targets for future theranostic applications. Current advancements in theranostics will undoubtedly necessitate changes in the training of nuclear medicine physicians. To better serve our clinical partners, it is essential to prioritize understanding picture acquisition and interpretation over the current emphasis on clinical skills and patient management, particularly in oncology [169]. On the other hand, the importance of combined nuclear medicine and radiology training for the education of imaging experts has increased. Nuclear medicine must establish itself as a distinct profession within the context of routine, diverse clinical practice. Therefore, it is necessary to enhance a distinct, independent profile that highlights specific advantages over similar professions (such as oncology, radiology, and radiation oncology), for example, by updating training program licensing [170].

Moreover, many businesses today view nuclear medicine theranostics as an intriguing business strategy. The recent acquisitions of the Advanced Accelerator Application (AAA) and Endocyte by Novartis are indicators of increasing business interest in theranostic development. The authorization of future radionuclide therapies will probably open up more opportunities, enabling a greater number of patients to receive well-tolerated targeted treatments using nuclear medicine.

### 3.1 Neuroendocrine tumours

In the COMPETE trial, 300 patients were randomly randomized to receive either 10 mg of everolimus daily or a maximum of four cycles of PRRT with 7.5 GBq of 177Lu-DOTATOC spaced three months apart. Overall survival was the secondary aim of the present study, and progression-free survival was the primary endpoint. After the failure of somatostatin analogues, the COMPETE trial is anticipated to result in wider usage of PRRT as the primary treatment for gastroenteropancreatic NETs [171]. A favourable response rate of 37% was discovered in a phase I therapeutic investigation using 68Ga/177Lu-DOTA-JR11, and after just one cycle of treatment, the size of the tumor significantly decreased. However, compared to somatostatin receptor agonists, this therapy also causes greater haematotoxicity [172,173].

### 3.2 PSMA-targeting therapy

In the therapy phase II trial, the second-line chemotherapeutic drug cabazitaxel was compared to 177Lu-PSMA-617 for treating mCRPC. 177Lu-PSMA radionuclide therapy (up to a maximum of 6 rounds of therapy) or cabazitaxel chemotherapy was administered randomly to 200 individuals with metastatic prostate cancer who progressed after hormonal therapy and first-line chemotherapy (up to a maximum of 10 cycles of therapy), and the severity of adverse events was examined in this trial [[174], [175], [176]]. In addition to PSMA-targeted radioligand therapy, this approach is another theranostic strategy that is being used more frequently for early-stage disease than for advanced prostate cancer recurrence. PSMA-targeting probes that produce radiation before surgery can be used to radioactively label tumor tissue [177].

### 3.3 Neurotensin/177Lu-3BP-227

The proliferation of tumor cells can be induced by the binding of neurotensin to NTSR1 via several signalling pathways, including the phospholipase C pathway. Moreover, activating kinases can increase the invasion and motility of tumor cells. For the management of tumours, neurotensin antagonists such as SR48692 have been developed [178,179]. Autoradio-radiographic studies have shown that the density of type 2 somatostatin receptors in NETs can reach the level of NTSR1 in pancreatic cancer. As a result, radiolabelled NTSR1 ligands are promising alternatives for pancreatic cancer radioligand therapy. An extremely potent nonpeptide NTSR1 antagonist is 177Lu-3BP-227 [[180], [181]].

## 4 Conclusion

Theranostics, a combination of therapy and diagnostics, has emerged as a promising approach in the field of oncology. Moreover, diagnostic tools can be used to identify specific molecular targets in tumours and subsequently deliver targeted therapies. This personalized medicine approach holds great potential for improving treatment outcomes and minimizing side effects in cancer patients.

In prostate cancer, theranostics have revolutionized the management of advanced stages of the disease. Targeted therapies that focus on PSMA, such as PSMA-based PET and PSMA-targeted radionuclide therapy, have shown promising results in detecting and treating prostate cancer lesions with high specificity. Theranostics for neuroendocrine tumours (NETs) have proven effective in both diagnosis and treatment. Gallium-68 DOTATATE PET imaging, which targets somatostatin receptors overexpressed in NETs, allows accurate tumor localization and staging. Peptide receptor radionuclide therapy (PRRT) utilizing radiolabelled somatostatin analogues has demonstrated significant clinical benefits in controlling tumour growth and improving patient survival. Breast cancer treatment also benefits from theranostics by targeting HER2. HER2-targeted imaging modalities, such as HER2-specific positron emission tomography (PET) or single-photon emission computed tomography (SPECT), enable precise detection of HER2-positive lesions. Combining HER2-targeted therapy with HER2-targeted radionuclide therapy has shown favourable results in preclinical and early clinical studies. Thyroid cancer theranostics have emerged as valuable tools for managing differentiated thyroid cancer (DTC). Radioiodine-based therapy, facilitated by diagnostic iodine-131 scanning, allows the identification of radioiodine-avid metastatic lesions. This approach helps tailor treatment strategies and optimize therapeutic response in patients with DTC. Gastrointestinal cancer theranostics have shown potential for treating gastrointestinal cancers, particularly colorectal cancer. Selective molecular imaging techniques, such as carcinoembryonic antigen (CEA)-targeted positron emission tomography (PET), enable accurate tumor detection and staging. Additionally, radiolabelled anti-CEA antibodies have been investigated as a targeted therapeutic option, delivering radiation specifically to CEA-expressing cancer cells.

In conclusion, theranostics has become a promising approach for the treatment of different cancers, enabling precise tumor detection, characterization, and targeted therapy delivery. By integrating diagnostics and therapeutics, theranostics have the potential to improve treatment outcomes, enhance patient care, and minimize unnecessary treatments. However, further research and clinical trials are desirable to validate the efficacy of this theranostic approach in different cancer types and explore its broader applications in personalized cancer medicine.

## Prior publication

None.

## Sources of support

None.

## Ethics statement

Review and approval from an ethics committee were not needed for this study because this was a literature review and because no new data were collected or analysed. For the same reason, informed consent was not needed.

## Grants and funding

None.

## Data availability

All the data are available here in this manuscript.

## CRediT authorship contribution statement

**Kuttiappan Anitha:** Writing – review & editing, Writing – original draft, Data curation, Conceptualization. **Santenna Chenchula:** Writing – review & editing, Writing – original draft, Visualization, Validation, Supervision, Conceptualization. **Vijayaraj Surendran:** Writing – review & editing, Software, Conceptualization. **Bhatt Shvetank:** Writing – review & editing, Supervision, Project administration. **Parameswar Ravula:** Writing – review & editing, Supervision, Software. **Rhythm Milan:** Writing – original draft, Visualization, Supervision. **Radhika Chikatipalli:** Writing – review & editing, Supervision. **Padmavathi R:** Writing – review & editing, Supervision.

## Declaration of competing interest

The authors declare the following financial interests/personal relationships which may be considered as potential competing interests:NONE If there are other authors, they declare that they have no known competing financial interests or personal relationships that could have appeared to influence the work reported in this paper.
